# Label-Free Study of the Global Cell Behavior during Exposure to Environmental Radiofrequency Fields in the Presence or Absence of Pro-Apoptotic or Pro-Autophagic Treatments

**DOI:** 10.3390/ijms23020658

**Published:** 2022-01-08

**Authors:** Alexandre Joushomme, André Garenne, Mélody Dufossée, Rémy Renom, Hermanus Johannes Ruigrok, Yann Loick Chappe, Anne Canovi, Lorenza Patrignoni, Annabelle Hurtier, Florence Poulletier de Gannes, Isabelle Lagroye, Philippe Lévêque, Noëlle Lewis, Muriel Priault, Delia Arnaud-Cormos, Yann Percherancier

**Affiliations:** 1 Univ. Bordeaux, CNRS, IMS/UMR 5218, F-33400 Talence, France; alexandre.joushomme@u-bordeaux.fr (A.J.); andre.garenne@u-bordeaux.fr (A.G.); remy330@msn.com (R.R.); hruigrok@sebia.com (H.J.R.); yann.chappe@u-bordeaux.fr (Y.L.C.); anne.canovi@u-bordeaux.fr (A.C.); lorenza.patrignoni@u-bordeaux.fr (L.P.); annabelle.hurtier@ims-bordeaux.fr (A.H.); florence.poulletier@ims-bordeaux.fr (F.P.d.G.); isabelle.lagroye@ephe.psl.eu (I.L.); noelle.lewis@u-bordeaux.fr (N.L.); 2 Univ. Bordeaux, CNRS, IBGC/UMR 5095, F-33000 Bordeaux, France; melody.dufossee@ibgc.cnrs.fr (M.D.); muriel.priault@ibgc.cnrs.fr (M.P.); 3 Paris Sciences et Lettres Research University, F-75006 Paris, France; 4 Univ. Limoges, CNRS, XLIM/UMR 7252, F-87000 Limoges, France; philippe.leveque@unilim.fr (P.L.); delia.arnaud-cormos@unilim.fr (D.A.-C.); 5 Institut Universitaire de France (IUF), F-75005 Paris, France

**Keywords:** label-free techniques, radiofrequency electromagnetic fields, bioelectromagnetics, digital holographic microscopy, impedancemetry, human cell lines, rat cortex primary cells, apoptosis, autophagy

## Abstract

It remains controversial whether exposure to environmental radiofrequency signals (RF) impacts cell status or response to cellular stress such as apoptosis or autophagy. We used two label-free techniques, cellular impedancemetry and Digital Holographic Microscopy (DHM), to assess the overall cellular response during RF exposure alone, or during co-exposure to RF and chemical treatments known to induce either apoptosis or autophagy. Two human cell lines (SH-SY5Y and HCT116) and two cultures of primary rat cortex cells (astrocytes and co-culture of neurons and glial cells) were exposed to RF using an 1800 MHz carrier wave modulated with various environmental signals (GSM: Global System for Mobile Communications, 2G signal), UMTS (Universal Mobile Telecommunications System, 3G signal), LTE (Long-Term Evolution, 4G signal, and Wi-Fi) or unmodulated RF (continuous wave, CW). The specific absorption rates (S.A.R.) used were 1.5 and 6 W/kg during DHM experiments and ranged from 5 to 24 W/kg during the recording of cellular impedance. Cells were continuously exposed for three to five consecutive days while the temporal phenotypic signature of cells behavior was recorded at constant temperature. Statistical analysis of the results does not indicate that RF-EMF exposure impacted the global behavior of healthy, apoptotic, or autophagic cells, even at S.A.R. levels higher than the guidelines, provided that the temperature was kept constant.

## 1. Introduction

In the last thirty years, thousands of in vitro studies have been conducted to assess the potential physiological impacts of exposure to radiofrequency (RF) and extremely low-frequency fields [1,2,3]. These studies showed how extremely low-frequency fields induce nerve stimulation and how high-level RF exposures cause tissue heating at the biophysical level. As a result, recommendations and standards have been established to protect populations from the associated health risks. Moreover, the potential non-thermal effects of low-level RF fields (i.e., at levels below the international guidelines) have been extensively studied without providing a definite answer based on physical knowledge. Two main challenges hinder the complete description of low-level RF effects: (i) the lack of knowledge about low-level effective field parameters and (ii) the complexity of the living machinery, making it challenging to define which cellular or molecular processes are targeted or impacted by RF exposure. In the context of health-risk assessment, this double challenge can only be addressed by continuing the search for the biological effects of RF exposure at levels below the guidelines using innovative methods.

Regarding co-exposures with environmental mutagens/carcinogens or chemical stressors such as pollutants, the situation is even more complex. For example, such co-exposures might affect DNA repair mechanisms, stimulate reactive oxygen species, or prevent free radical scavenging. Furthermore, one should still assess whether co-exposures between RF and known physical or chemical stresses lead to additive or synergistic adverse effects triggering cell death by apoptosis or autophagy [4,5].

The approaches previously used to study the cellular and molecular effects of low-level RF exposure have produced at best either large-scale data (such as “omics”) [6,7,8] or data acquired in real time and live cells, but focusing only on one cellular or molecular mechanism (e.g., the BRET approach that we developed recently [9,10,11]). Unfortunately, large-scale data are obtained from cell extracts following RF exposure, using, in most cases, stringent preparative steps. Such techniques help to identify cumulative stressor-triggered effects. Nonetheless, since cells exist in a metastable and often reversible equilibrium state, the risk is to overlook the RF effect due to the inability of these techniques to follow the dynamics of the living process. On the other hand, analyzing the dynamics of one specific cellular or molecular process in real-time under RF exposure requires knowledge of the RF interaction mechanism with living matter.

In summary, after decades of international research, whether RF exposure at levels below guidelines causes biological effects is still a matter of debate. Moreover, difficulties were encountered in interpreting and replicating the data of earlier studies, often acquired using ill-defined exposure conditions [2]. As a consequence, bioelectromagnetics research has stalled while societal concerns increased.

One solution to overcome these limitations is to use an empirical approach integrating the global cellular response during RF exposure or co-exposure with either chemicals or stressors. In the last fifteen years, label-free techniques emerged, offering the possibility of interrogating a broad panel of drug molecules with comprehensive coverage in targets, pathways, networks, and cellular processes of native cells in real-time. These techniques are based on several reading modes, such as cellular impedance measurement (also called cell impedancemetry), resonant waveguide grating, or digital holographic microscopy, and they detect morphological changes in cells challenged with chemicals [12,13,14]. Such techniques are powerful for revealing responses when there is little or no knowledge about the drug or stressor mechanism of action, and they are perfectly fit for bioelectromagnetics research. However, their use requires that cell-culture exposure to RF does not interfere with the read-out of the cell phenotype. To this aim, we first developed an EMF exposure device based on impedancemetry [15] to monitor in real time the phenotypic response of neuronal cells under RF exposure. Using this setup, we exposed cells to modulated- (GSM: *Global System for Mobile* Communications, 2G signal), UMTS (Universal Mobile Telecommunications System, 3G signal), LTE (Long-Term Evolution, 4G signal, Wi-Fi), or continuous-wave (CW) 1800 MHz signals at up to 24 W/kg over three consecutive days at a constant temperature. We then assessed the effect of RF exposure on the global behavior of SH-SY5Y neuroblastoma cells, which are often used as in vitro models of neuronal function and differentiation in bioelectromagnetics studies, and of both rat primary astrocytes and rat primary co-culture of neurons and glial cells. We also studied in cells the impact of combined exposures to RF and an environmental pollutant. Arsenic trioxide (As_2_O_3_) was chosen since arsenic is one of the most toxic metals derived from the natural environment. The current major cause of human arsenic toxicity is from contamination of drinking water from natural geological sources [16], and possibly partly upon the medical applications of arsenic trioxide (As_2_O_3_). Fortunately, the use of arsenical pesticides in agriculture has decreased dramatically [17]. In addition, various studies indicated that the actual molecular mechanism for As_2_O_3_-induced toxicity could be related to its triggering of apoptosis in many cell lines such as the SH-SY5Y cell line [18,19] and brain cells, including primary neurons [20] and glioma cells [21]. Finally, in a further effort, we also adapted a transverse electromagnetic cell (TEM cell) [22,23,24] onto a digital holographic microscope platform to assess whether CW or GSM-modulated 1800 MHz signals at up to 6 W/kg and steady temperature impacted the well characterized autophagic response of serum-deprived HCT116 cells [25].

## 2. Results

### 2.1. Temporal Signature of SH-SY5Y Cells Behavior in the Presence or Absence of As_2_O_3_ Using Impedancemetry

**Figure 1:**
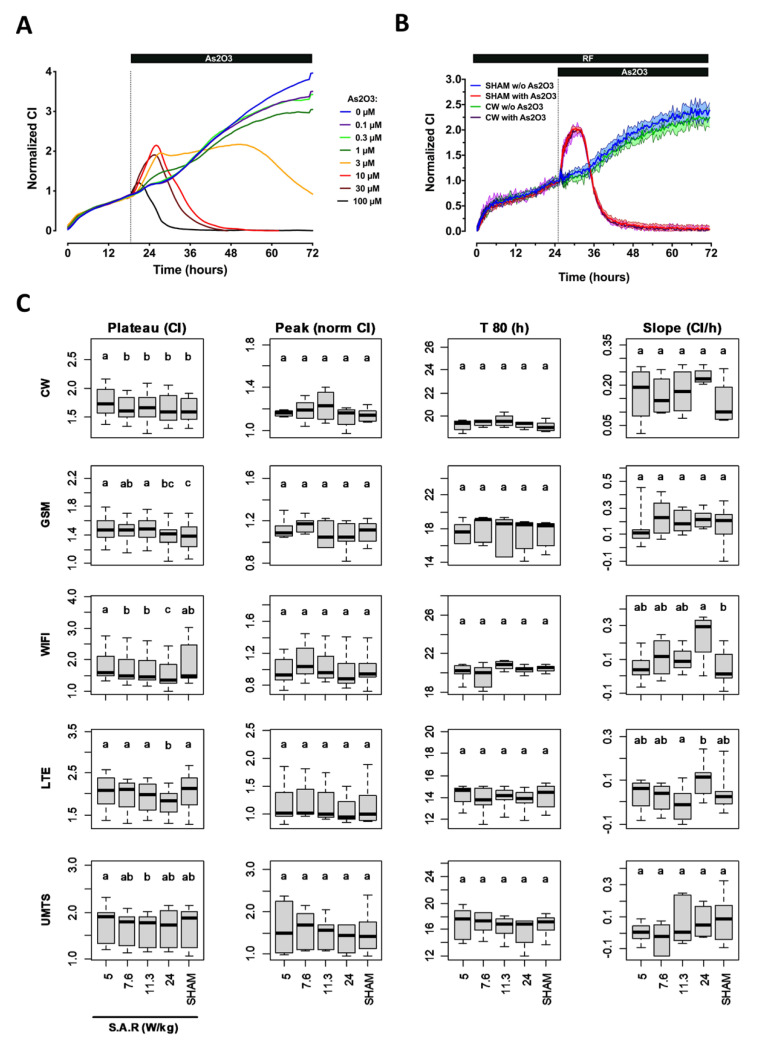
Characterization of the response of SH-SY5Y cells under RF exposure alone or under co-exposure to RF and As_2_O_3_. (**A**) Characterization of the normalized CI temporal signature of SH-SY5Y cells challenged with increasing concentrations of As_2_O_3_. The vertical dashed line indicates the time at which cells were either mock-challenged or challenged with the indicated concentration of As_2_O_3_. The data represent the average of four independent experiments. For better clarity, the measurement error is not represented. (**B**) Example of the CI temporal signature recorded from SH-SY5Y cell cultures that were either sham-exposed or exposed to unmodulated 1800 MHz (CW signal) at 24 W/kg. The vertical dashed line indicates the time at which cells were either mock-challenged or challenged with 10µM As_2_O_3_. The data represent the average of four independent experiments ± SEM values. (**C**) Statistical analysis of the effect of CW, GSM, Wi-Fi, LTE, and UMTS-modulated 1800 MHz signals emitted at either 5, 7.6, 11.3, or 24 W/kg on four metrics describing SH-SY5Y cells impedance measurement in the absence or presence of As_2_O_3_. First left column: average CI measured during the 5 h preceding injection of As_2_O_3_ or buffer. Second column to the left: magnitude of the CI increase in response to As_2_O_3_. Third column to the left: time at which 80% of the cell population died following As_2_O_3_ injection. Last column on the right: cell proliferation rate at day 3 in the absence of As_2_O_3_. The number of replicates per condition is indicated in Supplementary Table S1.

Using our already described xCELL-RF system [15], we first assessed the temporal signature of the impedance variation of SH-SY5Y neuroblastoma cells over 72 h (Figure 1 A&B, blue curve). The impedance variation is here defined by the Cell Index variable (CI, see Material and Methods). Right after cell seeding, cells started to adhere, thereby inducing a rapid increase of the CI value. The adhesion phase was followed by a first pseudo-plateau during which cells are spreading and preparing for dividing. Finally, 24 h after seeding, cells started to grow exponentially, as indicated by a continuous increase in the CI value. Such temporal signature agrees with data by other groups for proliferating cells [26].

We then assessed the impact of As_2_O_3_ on the temporal signature measured with SH-SY5Y neuroblastoma cells. Eighteen hours after seeding, increasing concentrations of As_2_O_3_ were added to the culture media, and impedance was recorded during the following 54 h (Figure 1A). Immediately after As_2_O_3_ injection, we observed a complex biphasic response which shape depended on the concentration of As_2_O_3_. For the two lowest concentrations of As_2_O_3_ tested (0.1 µM and 0.3 µM), the temporal signature of the CI variation followed closely the one obtained with the control group, with a slightly smaller CI at the end of the experiment. When 1 µM of As_2_O_3_ was added, we measured a slight increase in the relative CI followed by a pseudo-plateau that lasted several hours before cells started to proliferate, and an even more pronounced decrease in the CI at the end of the experiment. Above 1 µM of AS_2_O_3_, the temporal signature recorded in SH-SY5Y cells behaved as a bell-shaped curve which magnitude peaked at 10 µM. When using 3 µM of As_2_O_3_, the bell-shaped response was followed by a long plateau lasting approximately 28 h before the CI started to decrease. Above 3 µM As_2_O_3_, such a plateau no longer occurred. At these higher concentrations of As_2_O_3_, after an initial increase (primary response), the CI gradually decreased to the background (secondary response). This decrease in CI indicates that cells are dying due to the toxicity of As_2_O_3,_ which causes them to detach from the gold electrode array. The onset of this decrease appeared more rapidly as the concentration of As_2_O_3_ increased. Overall, the complex temporal profile of the CI variation indicates that As_2_O_3_ above 1 µM elicited a primary response in SH-SY5Y cells with an immediate increase in the CI. The fate of the cell culture then relies on the biochemical reactions elicited during this primary response with a switch between cell survival and cell death at around 2 µM As_2_O_3_.

### 2.2. Effect of RF Exposure on SH-SY5Y Cell Behavior Assessed by Impedancemetry in the Presence or Absence of As_2_O_3_ Co-Exposure

We next assessed whether exposure to various 1800 MHz signals (CW, GSM, Wi-Fi, UMTS, and LTE) at S.A.R. of 5, 7.6, 11.3, and 24 W/kg impacted the temporal signature of the SH-SY5Y cells recorded in the absence or presence of 10 µM As_2_O_3_. This concentration was chosen since it induced an intermediate temporal signature that allows the detection of either a positive or a negative effect of RF exposure on the As_2_O_3_ efficacy to induce cell death in SH-SY5Y cells (Figure 1A). RF exposure started immediately after the cells were seeded in the wells of the xCELLigence plates, and continued throughout the experiment.

As exemplified in Figure 1B, there was no qualitative difference between the temporal signatures recorded from sham-exposed cells or cells exposed to a CW 1800 MHz RF signal, whatever SH-SY5Y cells were treated with As_2_O_3_ or mock-treated. Therefore, to quantitively characterize the potential effect of RF alone or in co-exposure with As_2_O_3_, we defined four metrics: (i) the relative quantity of cells that successfully attached and spread onto the interdigitated electrode array as measured by the average CI of the plateau phase acquired during the 5 h preceding injection of As_2_O_3_ or buffer (metric 1), (ii) the magnitude of the CI increase in response to As_2_O_3_ (metric 2), (iii) the time moment at which 80% of the cell population was dead following As_2_O_3_ injection (metric 3), and (iv) the cell proliferation rate obtained by measuring the slope of the growth phase recorded in the absence of As_2_O_3_ (metric 4). As shown in Figure 1C, we only measured minors but significant differences between sham and some exposed conditions when considering metric 1 (e.g., Wi-Fi and LTE signals on SH-SY5Y cells). The only other difference measured between sham- and exposed-conditions concerned a slight increase of SH-SY5Y cell proliferation rate in the absence of As_2_O_3_ (metric 4) under exposure to a Wi-Fi signal at 24 W/kg.

### 2.3. Effect of RF Exposure on Cortical Astrocytes and Neuron-Glia Co-Culture Cell Behavior Assessed by Impedancemetry in the Presence or Absence of As_2_O_3_ Co-Exposure

**Figure 2:**
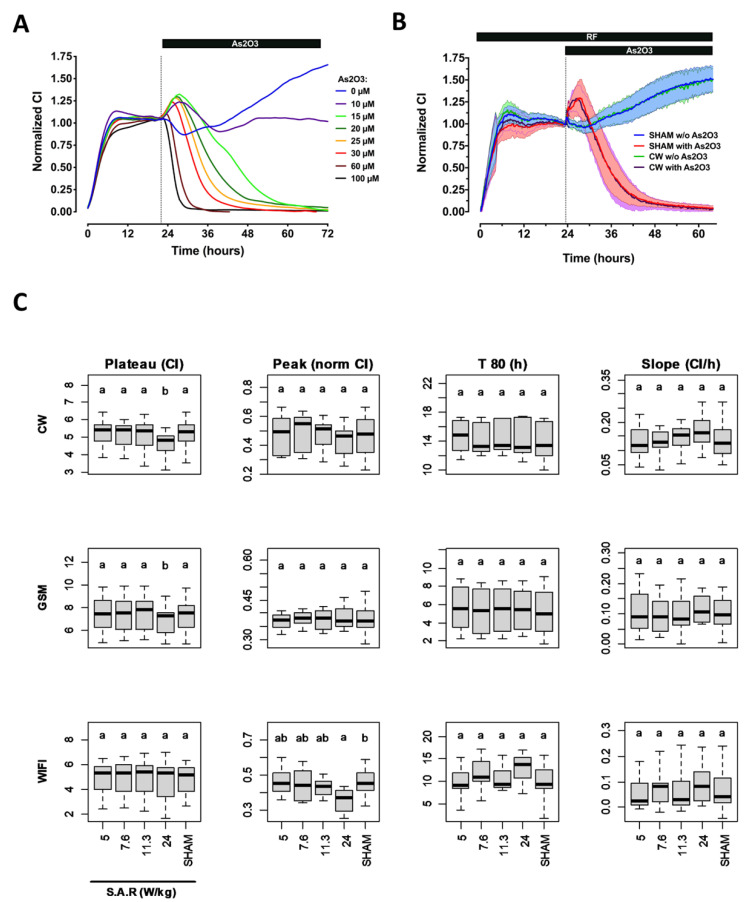
Characterization of the response of primary astrocytes cells under RF exposure alone or under co-exposure to RF and As_2_O_3_. (**A**) Characterization of the CI temporal signature of primary rat astrocytes cells challenged with increasing concentrations of As_2_O_3_. The vertical dashed line indicates the time at which cells were either mock-challenged or challenged with the indicated concentration of As_2_O_3_. The data represent the average of three independent experiments. For better clarity, the measurement error is not represented. (**B**) Example of the normalized CI temporal signature recorded from primary astrocytes cell cultures that were either sham-exposed or exposed to unmodulated 1800 MHz (CW signal) at 24 W/kg. The vertical dashed line indicates when cells were either mock-challenged or challenged with 30 µM As_2_O_3_. The data represent the average of 9 to 11 independent experiments ± SEM values. (**C**) Statistical analysis of the effect of CW, GSM, and Wi-Fi-modulated 1800 MHz signals emitted at 5, 7.6, 11.3, or 24 W/kg on four metrics describing primary astrocyte cells impedance measurement in presence or absence of As_2_O_3_. First left column: average CI measured during the 5 h preceding injection of As_2_O_3_ or buffer. Second column to the left: magnitude of the CI increase in response to As_2_O_3_. Third column to the left: time at which 80% of the cell population died following As_2_O_3_ injection. Last column on the right: cell proliferation rate at day 3 in the absence of As_2_O_3_. The number of replicates per condition is indicated in Supplementary Table S1.

**Figure 3:**
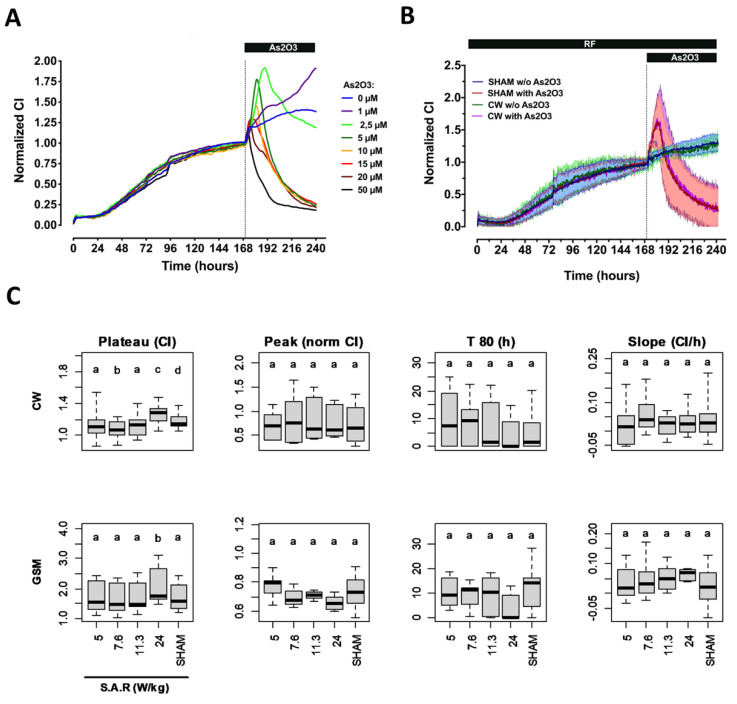
Characterization of the response of primary cortical cells co-culture under RF exposure alone or co-exposure to RF and As_2_O_3_. (**A**) Characterization of the CI temporal signature of primary cortical cells co-culture challenged with increasing concentrations of As_2_O_3_. The vertical dashed line indicates the time at which cells were either mock-challenged or challenged with the indicated concentration of As_2_O_3_. The data represent the average of three independent experiments. For better clarity, the measurement error is not represented. (**B**) Example of the normalized CI temporal signature recorded from primary cortical cells co-culture that were either sham-exposed or exposed to unmodulated 1800 MHz (CW signal) at 24 W/kg. The vertical dashed line indicates when cells were either mock-challenged or challenged with 5 µM As_2_O_3_. The data represent the average of 5 to 6 independent experiments ± SEM values. (**C**) Statistical analysis of the effect of CW or GSM-modulated 1800 MHz signals emitted at 5, 7.6, 11.3, or 24 W/kg on four metrics describing primary cortical cells co-culture impedance measurement in absence or presence of As_2_O_3_. First left column: average CI measured during the 5 h preceding injection of As_2_O_3_ or buffer. Second column to the left: magnitude of the CI increase in response to As_2_O_3_. Third column to the left: time at which 80% of the cell population died following As_2_O_3_ injection. Last column on the right: Cell proliferation rate during the last day of the experiment in the absence of As_2_O_3_. The number of replicates per condition is indicated in Supplementary Table S1.

We next wanted to assess the potential effect of RF on the global behavior of primary astrocytes (Figure 2) and mixed cortical neuron-glia co-cultured cells (Figure 3). These cells are considered the ones most at risk when a cell phone is in the head proximity. Neonatal rats’ cortex astrocytes and a neuron-glia co-culture were processed as the SH-SY5Y neuroblastoma cells. We first assessed whether the CI temporal variation of these primary cells was similar to the one observed with the SH-SY5Y cell line. Following the seeding of astrocytes in the xCELLigence plate wells, the CI increased rapidly until reaching a plateau approximatively 10 h after the beginning of the experiment. At 22 h postseeding, astrocytes were then challenged with increasing concentrations of As_2_O_3_ or mock challenged, and the CI variation was measured up to 72 h postseeding (Figure 2A). As observed for the SH-SY5Y cells, As_2_O_3_ injection induced a rapid and immediate bell-shaped variation of the CI index, with an initial increase (primary response) followed by a dramatic CI drop that decreased to zero for all concentrations tested except the lowest one (10 µM). At that concentration, the magnitude of the primary response was already close to maximal, and cell growth resumed compared with the mock-activated condition. Above 10 µM, the primary response’s magnitude and length inversely varied with the increase in As_2_O_3_. The highest and longest primary response was obtained with 15 µM of As_2_O_3_. Above 15 µM, both magnitude and duration of the primary response decreased while cells died more rapidly, as indicated by an early drop of the CI when As_2_O_3_ concentration increased.

Assessing the temporal signature of neurons and glial cells co-culture needed longer experiments since the CI was very low during the initial plateau phase that followed cell seeding. At 24 h postseeding, the cortical cell culture showed a continuous increase in the CI due to the proliferation of glial cells [27] (Figure 3A). Culture media was thus refreshed 90 h postseeding, and As_2_O_3_ was injected in the culture media 170 h post seeding. Injection of As_2_O_3_ again induced a complex response with an increase in cell culture growth rate at the lowest concentration tested (1 µM) and a bell-shaped response above 1 µM. The magnitude of the primary response was maximal at 2.5 µM and then decreased with the increase in As_2_O_3_ concentration. This decrease of the primary response to As_2_O_3_ is due to a sooner onset of the CI decrease, indicating that increasing As_2_O_3_ concentration accelerated cell death. In sharp contrast with what was observed on SH-SY5Y and astrocytes cells, we did not measure any difference in the shape of the CI decrease, except for the highest concentration of As_2_O_3_.

Based on these concentration-response data, we used As_2_O_3_ concentrations of 5 and 30 µM, to trigger an intermediate response within the cortical cells co-culture and the astrocytes culture respectively. Of note, mock activation induced a moderate and transient change of the cortical cells co-culture temporal signature, indicating that physical constraints such as shear stress can modify cortical cell growth or behavior. Having characterized the temporal signature of the CI variation in astrocytes and cortical cells co-cultures in the presence or absence of a suboptimal concentration of As_2_O_3_, we assessed whether RF exposure modified such signals. As shown in Figure 2B and Figure 3B for both primary cell cultures, whether or not cells were challenged with As_2_O_3_, no qualitative differences were shown when comparing the temporal signature of the CI variation in cells exposed to 1800 MHz CW signal at 24 W/kg and in sham-exposed cells. This was confirmed by the lack of differences between RF and sham conditions for almost all metrics considered (Figure 2C and Figure 3C). We only measured a small but significant variation of the CI reached before As_2_O_3_ or mock injection (metric 1) when astrocytes and cortical cell co-cultures were exposed to CW or GSM-modulated 1800 MH signals at 24 W/kg. Under those conditions, the CI decreased slightly in RF-exposed astrocytes and increased slightly in RF-exposed cortical cell co-culture. This parameter was not impacted when astrocyte cells were exposed to Wi-Fi-modulated 1800 MHz signals, whatever S.A.R. was used. We also measured a slight decrease of the primary response magnitude (metric 2) following As_2_O_3_ treatment of astrocytes cells exposed to Wi-Fi signal.

### 2.4. Characterization of Serum Starvation-Induced Autophagy in HCT116 Colon Cancer Cells Using Digital Holographic Microscopy

**Figure 4:**
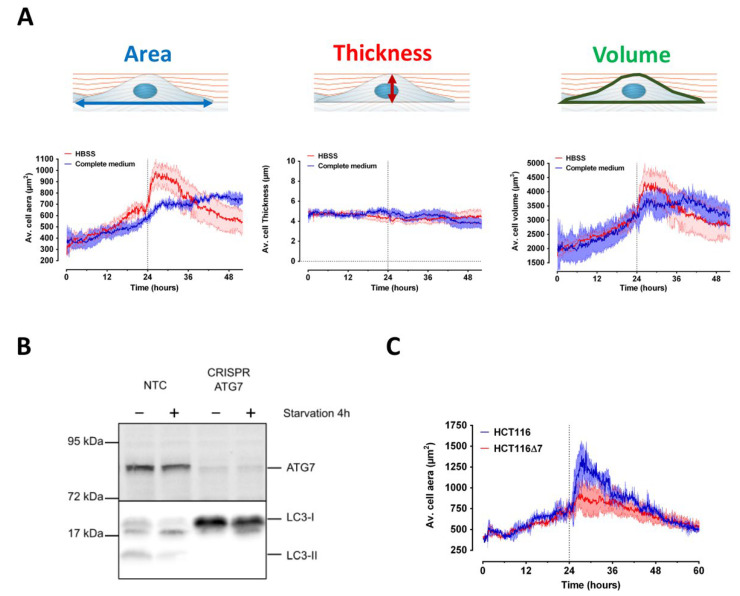
Effect of serum deprivation-induced autophagy on HCT116 cells area, thickness, and volume assessed by DHM. (**A**) HCT116 cells were left in culture for 24 h before the culture medium was either refreshed or replaced with HBSS serum-free medium at the time indicated with a vertical dashed line. DHM measurement of HCT116 cells area (left column), thickness (middle column), or volume (right column) was performed throughout the experiment that lasted up to 55 h. The data represents average of three to four independent experiments ± SEM values. (**B**) Functional validation of autophagy-deficient HCT116 cells: HCT116 cells transduced with non targeting sg-RNA (NTC) or with ATG7 sg-RNA (CRISPR-ATG7) were grown in complete medium or transferred to HBSS for 4 h (serum deprivation induced autophagy). The efficiency of ATG7 gene edition was verified by western blot on the top panel (Cell Signaling Rabbit monoclonal antibody D12B11). The absence of lipidation of the autophagy protein LC3-I into PE-conjugated LC3-II in HCT116-CRISPR-ATG7 cells confirms that autophagy was functionally impaired in these cells compared to NTC cells (bottom panel—Sigma anti-LC3B rabbit antibody L7543). (**C**) HCT116 or HCT116-CRISPR-ATG7 cells were left in culture for 24h before the culture medium was replaced with HBSS serum-free medium at the time indicated with a vertical dashed line. Cells area was measured throughout the experiment using DHM. The data represents the average of three independent experiments ± SEM values.

In a complementary label-free approach, we combined a TEM cell, previously adapted for microscopy [22,24], with digital holographic microscopy (DHM) to assess whether RF exposure could impact cell autophagy (see Material and Methods). Thanks to this setup, the morphology of HCT116 cells, a well-known model for the study of autophagy induced by serum-deprivation [25], was first assessed in absence of RF exposure (Figure 4). The evolution of various parameters such as cells thickness, volume and contact area of cells with culture plate were derived from the DHM images. As shown in Figure 4A, both HCT116 cells average area and volume slowly increased over time reaching a plateau at approximatively 28 h after the beginning of the experiment. Refreshing the cell culture media (control condition with complete medium) did not induce any detectable perturbation in the average cell volume and area temporal evolution. Interestingly, the thickness of the cells varied only once the culture medium was refreshed, decreasing slightly with a wave-like pattern. Replacing the Dulbecco’s modified Eagle’s medium (DMEM) complete culture medium (see Material and Methods for culture conditions) with serum-free Hanks’ Balanced Salt Solution (HBSS), a treatment known to induce autophagy in HCT116 cancer colon cells [25], elicited an immediate response which led to a significant change in cell morphology (Figure 4A). During the first 6 h following serum deprivation, the cell area increased dramatically compared to the control condition. Then, after reaching a peak, the cell area suddenly decreased progressively to a value lower than that measured under control conditions. The temporal evolution of the average cell volume followed a similar although less marked pattern. The average cell thickness remained, however, unchanged after serum deprivation.

To assess whether the initial increase in the average cell area is indicative of an autophagic response, we generated the HCT116-CRISPR-ATG7 cell line that does not express the ATG7 gene (Figure 4B), which is mandatory to promote autophagy [28]. As expected, ATG7 knock-out rendered the HCT116-CRISPR-ATG7 cell line deficient for autophagy as demonstrated by the abrogation of LC3-I/II conversion following serum deprivation by replacement of the DMEM culture medium by HBSS buffer (Figure 4B). As shown in Figure 4C, in contrast to the wild-type HCT116 cells, the initial increase of the average cell area measured during the first six hours following serum deprivation was abolished in HCT116-CRISPR-ATG7 cells. Instead, serum deprivation induced a slow decrease of the HCT116-CRISPR-ATG7 average cell area which continued until the end of the recording, reaching a similar value to the control cell line. This last result indicates that the autophagic process in serum-deprived HCT116 cells is accompanied by an increase in the cell area in contact with the cell culture plate. Cell volume also increased during the first six hours following serum deprivation while the thickness of the cells remained constant. The cell area decrease observed after this initial response likely reflects the cell failure to overcome the absence of serum and the subsequent cell death. The median value of the cell area measured during the last 3 h before serum deprivation, the relative magnitude of the autophagic response during the 3 h following serum deprivation, and the cell death rate in the last 20 h of the experiments represent therefore good indicators for quantitatively evaluating the HCT116 cell signature in our experiments.

### 2.5. Effect of RF Exposure on the Autophagic Response of HCT116 Cells

**Figure 5:**
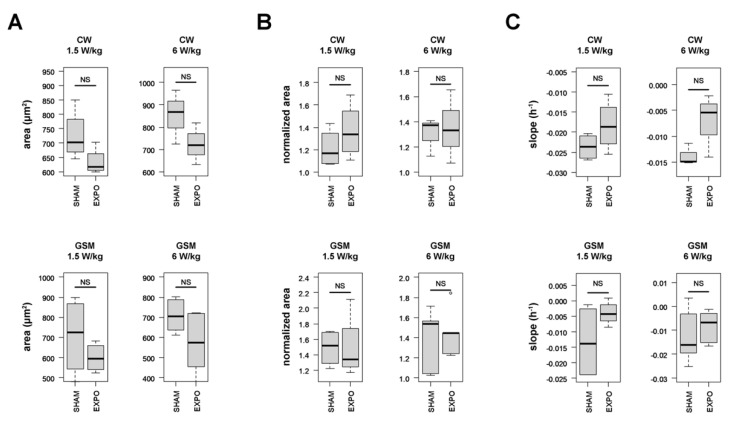
Statistical analysis of the effect of CW (upper panels) and GSM (lower panels)—modulated 1800 MHz signals emitted at either 1.5 or 6 W/kg on the average cell area measured during the last four hours before serum deprivation (**A**), the relative magnitude of the autophagic response during the 6 h following serum deprivation (**B**), and the cell death rate in the last 20 h of the experiments (**C**). Three to four biological replicates were performed for each experimental condition. NS: the difference is not significant between ”SHAM” and “EXPO” conditions.

We then assessed the impact of RF exposure on autophagy in HCT116 cells. The cells were sham-exposed or exposed to CW or GSM-modulated 1800 MHz signals at 1.5 and 6 W/kg, two levels that are slightly below and above the guidelines, respectively. The average cell area was measured in real-time under sham or RF exposure before and after serum deprivation, which occurred 24 h after the beginning of RF exposure. Whatever the signal considered and whatever the specific absorption rate (S.A.R.) considered, we could measure a slight decrease of both the median cell area measured during the last 3 h before serum deprivation (Figure 5A) and the cell death rate during the last 20 h of the experiment (Figure 5C). Nonetheless, none of these effects reached statistical significance. The magnitude of the autophagic response following serum deprivation remained unchanged under RF exposure (Figure 5B).

## 3. Discussion

It remains controversial whether exposure to environmental radiofrequency signals impacts cell status or response to cellular stress such as apoptosis or autophagy. We here took advantage of the impedancemetry and holographic microscopy techniques to assess the overall cellular response during RF exposure alone or during co-exposure to RF and chemical treatments known to induce either apoptosis or autophagy.

Regarding the cell types used in our study (e.g., SH-SY5Y human glioblastoma cell line, HCT116 human colon cancer cells, rat primary astrocytes and rat primary neurons—glia co-culture), many research groups have evaluated the potential impact of RF signals on cell proliferation or cell death, including apoptosis and autophagy.

Ten studies reported experiments in which cell proliferation and/or cell death were studied in SH-SY5Y cells exposed to RF-EMF [29,30,31,32,33,34,35,36,37,38]. Half of these studies exposed SH-SY5Ycells to RF-EMF in the 872–935 MHz band, while the other studies were in the 1760–2100 MHz band. In addition, half of the studies focused on CW or GSM-modulated signals, while the others used pulsed, intermittent, W-CDMA, or LTE modulated signals. The S.A.R. levels ranged from 0.086 W/kg [38] to 5W/kg [34], and the duration of exposure was from several minutes [35] to 3 days [32]. Eight studies reported no effect of RF-EMF exposure on apoptosis [29,30,31,38], cell proliferation [33,34], cell viability [36], or cell differentiation [37]. Only two studies reported that RF-EMF exposure impacted SH-SY5Y cell proliferation after a 24 h exposure to a GSM modulated 900 MHz signal at 1 W/kg [35] or after a 72 h exposure to an LTE-modulated 1760 MHz signal at 2 W/kg [32]. Of note, two studies from the Scarfi group described that SH-SY5Y cells previously exposed for 20 h to an UMTS-modulated 1950 MHz signal at 0.3 W/kg developed an adaptive response to subsequent treatment with menadione [39,40]. While in our study we also exposed SH-SY5Y cells to RF for a similar duration before challenging cells with As_2_O_3_, we did not measure any effect related to an adaptive response in our experimental conditions. Both studies cannot however be compared since we used a very different RF signal (in terms of carrier wave frequency, S.A.R., and modulation) and since we challenged cells with As_2_O_3_ instead of menadione. Whether our xCELL-RF setup could help investigating an RF-induced adaptive response in SH-SY5Y cells or other cell types needs to be evaluated in future studies.

Two research articles described the effect of various RF signals on human colon cancer HCT116 cells. First, the impact of high power and short duration oscillatory pulses of electromagnetic fields with a signal frequency of either 150 or 1500 MHz on various biological parameters, including cell growth and apoptosis, was investigated by Gibot et al. (2019) without showing any significant effect [41]. More recently, Ozgur et al. (2021) showed increased apoptosis and inflammatory response in HCT116 cells exposed for 1 and 4 h to an intermittent (15 min on / 15 min off) GSM-EDGE modulated signals at 900, 1.800, and 2.100 MHz at 2 W/kg [42]. However, no numerical dosimetry was performed in this research work, and the temperature of the culture medium during exposure was not monitored, casting doubt on the occurrence of a non-thermal effect.

Regarding the effects of RF on primary cell cultures from brain origin, nine articles reported the effects of 900, 1800, and 1950 MHz RF signals on neonatal cortical cells [43,44,45,46,47], microglial cells and astrocytes [46,48,49], embryonic neural stem cells [50,51], and spiral ganglion neurons [48]. Six of these studies focused on GSM-modulated signals, while the others used either a CW signal or a TD-SDMA-modulated signal. The S.A.R. used ranged from 0.25 W/kg [46,50,51] to 5.36 W/kg [49] and the duration of exposure ranged from 15 min [51] to 3 days [50]. Six studies reported no effects of RF exposure on cell proliferation, cell viability, apoptosis, or autophagy [43,46,47,48,50,51]. A small but significant increase in autophagy could be measured when spiral ganglion neurons were co-exposed to lipopolysaccharide and an intermittent (5 min on/10 min off) GSM-modulated 1800 MHz signal emitted at 4 W/kg for 24 h [48]. A decrease in neonatal cortical cells viability was measured by Zhu et al. (2008) after exposure of the ex-vivo cell culture to a 900 MHz signal at 0.1 mW/cm^2^ [44]. Unfortunately, in this last article, the exposure system was ill-defined and the modulation of the signal was not indicated, preventing any conclusion. In the study by [51], differentiation was highly diminished in neural stem cells but not in astrocytes, following exposure to a GSM-modulated 900 MHz signal emitted for 2 h at a 2.287 W/kg using a GSM mobile phone simulator. Again, the lack of proper characterization of the exposure system precluded any conclusion regarding the reported effects. In the study by Joubert et al. (2008), an increased apoptosis rate was measured in rat primary cortical neuronal cultures exposed to 900 MHz CW RF fields for 24 h at 2 W/kg [45]. Interestingly, this effect was more pronounced 24 h after exposure. The authors indicated that, despite state-of-the-art dosimetry of their experiments and assessment of temperature bias, thermal effects of the RF field at the interface between the culture medium and the support could not be ruled out. In the study by Liu et al. (2012), a marked decrease in cell proliferation, increased cell apoptosis, and morphological changes in the cells were detected in mouse primary astrocytes after 48 h of exposure to 1950-MHz TD-SCDMA at 5.36 W/kg [49]. The RF-EMF were emitted from a calibrated standard dipole, but no numerical dosimetry of the exposure was described. Moreover, no details were given about the temperature regulation of the cell plate. Therefore, it might be possible that local thermal effects caused the cells to undergo apoptosis.

Overall, most of the studies herein discussed have shown no effects on apoptosis, autophagy, or cell proliferation following in vitro exposure of either SH-SY5Y and HCT116 cell lines or primary culture from rodent brain. Furthermore, many of the studies showing an effect are questionable in terms of exposure condition characterization, which casts doubts on the reports of a pure non-thermal effect. Some research groups have emphasized the negative association between the outcome of cellular response in bioelectromagnetics studies and the quality of the experimental procedures, particularly the exposure conditions [52]. Nonetheless, many studies mentioned above relied on endpoints measured after various exposure durations. Consequently, such studies may have missed a transient effect due to RF exposure.

In our study, both impedancemetry and holographic microscopy techniques gave access to the temporal variation of different endpoints in cell cultures under RF exposure, thus precluding to overlook any transient effect of RF exposure. Using impedancemetry, any response that induces changes in cell morphology (size, volume, shape, or spreading), cell number (proliferation or death), or movement (migration or extravasation) are investigated through the monitoring of a unique index. The setup design allows simultaneous measurement of multiple wells, including sham-exposed and RF-exposed cells at four different S.A.R. Digital holographic microscopy gives access to a more precise analysis of the cell phenotype since, in addition to cell proliferation, we can measure about twenty different parameters in real-time, including cell volume, length, and area. However, we measured only one sample per microscope under sham or RF exposure.

Using impedancemetry, we measured the effects of five signals (CW, GSM, Wi-Fi, UMTS, and LTE) on SH-SY5Y cells, three different signals on astrocytes (CW, GSM, and Wi-Fi), and two signals on cortical neurons and glial cells (CW and GSM). The exposure lasted 70 h for SH-SY5Y and astrocytes and 110 h for co-cultured cortical neurons and glial cells. Each time, the effect of a given signal was measured simultaneously at four levels above the guidelines (5, 7.6, 11.3, and 24 W/kg) in addition to the sham condition. All assays were performed in the absence or presence of a suboptimal concentration of As_2_O_3_ to assess both the effects of RF alone and a potential effect of co-exposure to RF with a chemical stressor. Consequently, 110 different experimental conditions were tested. We measured four different metrics on the acquired temporal signatures, that describe the cell behavior at critical time points and give insights into how RF could have impacted (i) cells adherence and spreading, (ii) the capacity of the cells to respond to As_2_O_3_, (iii) cell death rate following As_2_O_3_ injection, and (iv) cell proliferation rate in the absence of As_2_O_3_. Our results indicate that, whatever the RF signal, S.A.R., or the cell type considered, there is almost no difference in the capacity of the cells to respond to As_2_O_3_ (metrics 2 and 3) and in cell proliferation rate (metric 4). We only measured a ~20% decrease in the amplitude of the initial CI increase occurring following As_2_O_3_ injection (primary response) when astrocytes were exposed to a Wi-Fi signal at 24 W/kg, and an increase of the cell proliferation rate when SH-SY5Y cells were exposed also to Wi-Fi signal at 24 W/kg. Slight but statistically significant variations were however measured for cell attachment and cell spreading (metric 1) between sham and exposed conditions, especially at 24 W/kg, a value significantly higher than the guidelines. This can be due to an increase in the metallic losses of the electrodes. Whatever the experimental conditions considered, no transient effect of RF exposure on the temporal signature of both resting and As_2_O_3_-challenged cells was observed.

In a further effort to unravel potential RF effects on resting and stressed cells, we also used DHM to study the effect of CW or GSM-modulated 1800 MHz RF-EMF, at 1.5 and 6 W/kg, on HCT116 colon cancer cell lines. We selected this cell line since it is a well-known model to study autophagy in serum deprived conditions [25]. We first assessed whether DHM was prone to detect phenotypic changes in autophagic cells following serum deprivation. Using DHM, we measured the average HCT116 cell volume, thickness, and area before and after serum deprivation. All these parameters are informative to discriminate between apoptosis and necrosis, the two different types of cell death, but nothing has been described so far for autophagy [53]. According to Alm et al. (2013) [53], a decrease in cell area with a slight increase in volume and thickness occur at the onset of the apoptosis process. Following this first transient response, the thickness of cells increases gradually until reaching a plateau and finally decreases, while both cells area and volume concomitantly and continuously decrease. This indicates that cells basis shrinks during apoptosis while the cells bodies stretch and fragment. The temporal signature of necrosis is slightly different with a decrease of both thickness and volume but with an increase in cells area. This indicates that during necrosis, contrary to apoptosis, the cells spread out and flatten while dying [53]. We here complete the observations of Alm et al. [53] by describing the temporal signature of autophagy. Autophagy is characterized by a first transient response where both the average cell areas and volumes transiently and rapidly increase following serum privation. From the molecular standpoint, autophagy onset in response to stress is known to be very fast, as it only takes 20 min to form functional autophagosomes. Our study analyses autophagy from the morphological standpoint, and shows that three-to-four hours following serum deprivation, both average cell areas and volumes reach a plateau and then continuously decrease until the end of the experiment. During this process, the thickness of the cells remains almost identical, indicating that the basis of the autophagic cells enlarges before contracting. Notably, the first transient response (an increase in average cell volume, characterized by a broader cell basis and no change in cell thickness) was not observed in HCT116-CRISPR-ATG7 autophagy-deficient cells, thereby showing the specificity of our read-out. After this first period, the time signatures for HCT116 WT and HCT116-CRISPR-ATG7 overlap, probably indicating that the autophagic process did not compensate for the lack of nutrients, leading to cell death.

In a second step, we assessed the impact of RF fields on healthy and autophagic cells by measuring the cell area which was the lead parameter for assessing the autophagic response. Wild-type HCT116 cells were continuously exposed under CW or GSM-modulated 1800 MHz signals at 1.5 and 6 W/kg for 24 h before autophagy was triggered by serum deprivation. In addition, the temporal signature of both sham and RF-exposed HCT116 cells was measured for a further 36 h. Quantitative analysis of the temporal signature revealed that RF exposure, whatever the signal or the S.A.R. considered, slightly decreased both average cell area before serum deprivation and cell death rate in the last 20 h of the experiments, with no change in the autophagic response. Such difference nonetheless did not reach statistical significance.

Overall, our time-resolved analysis under experimental conditions where the temperature was kept constant did not show any effects of RF on the cellular response in healthy, apoptotic, or autophagic cells, even at S.A.R. levels above the international guidelines. Further studies complying with rigorous and standardized experimental methodology [52,54] will help consolidate our findings.

## 4. Materials and Methods

### 4.1. Reagents

Arsenic trioxide (ATO, As_2_O_3_) was acquired from Sigma-Aldrich, St. Louis, MO, USA.

### 4.2. Cell Culture

Undifferentiated SH-SY5Y cell line (ATCC, Cat. No. CRL2266, Manassas, VA, USA) cells were cultured in Dulbecco’s minimal essential medium: Nutrient Mixture F-12 (DMEM/F12) with 15% fetal bovine serum, 1% non-Essential Amino Acids supplemented with 1% penicillin-streptomycin (Thermo Fisher Scientific, Waltham, MA, USA).

HCT116 human colorectal carcinoma cells were initially obtained from Dr. Vogelstein (Johns Hopkins University, Baltimore, MD, USA). Using the lentiCRISPR system detailed in O’Prey et al. (2017) [55], HCT116 cells deleted for ATG7, and therefore deficient for autophagy (noted HCT116-CRISPR-ATG7) were generated. Both HCT116 and HCT116-CRISPR-ATG7 cells were maintained in Dulbecco’s modified Eagle’s medium-high glucose (DMEM, Cat. No. D6429; Sigma-Aldrich, St. Louis, MO, USA) supplemented with 10% fetal bovine serum, 100 units ml^−1^ penicillin, and streptomycin.

The primary neuronal cells were obtained from the cortex of embryonic (E18) Sprague–Dawley rats (Charles River Laboratories, L’Arbresle, France). After 5% isoflurane anesthesia, the gestating rat was sacrificed by elongation. Next, embryo cortices were dissected in Dulbecco’s Modified Eagle Medium (DMEM)-penicillin/streptomycin (Fisher Scientific, Waltham, MA, USA) and treated for 30 min with an enzymatic solution containing 20 units ml^−1^ of papain and 0.005% of DNase (Worthington Biochemical Corporation, Lakewood, CO, USA). The fragments were then subjected to mechanical dissociation using a 10-mL serological pipette and centrifuged at 300× *g* for 5 min at room temperature. Next, the supernatant was eliminated, and the pellet was resuspended in a solution with DNase. This latter mixture was placed above an albumin-inhibitor solution to create a discontinuous density gradient and then centrifuged at 70× *g* for 6 min at room temperature. In this last step, the dissociated cells (in the pellet) were separated from membrane fragments of dead cells in the supernatant. Finally, the pellet containing cortical cells was suspended in the culture medium composed of neurobasal medium supplemented with 2% B-27, 1% GlutaMAX, and 1% penicillin/streptomycin (Fisher Scientific). Each well was plated with a suspension of 200,000 cells in 100 µL. Laminin (L2020, Sigma–Aldrich, St. Quentin-Fallavier, France) was used at the concentration of 50 µg/mL to coat xCELLigence wells 3 h before seeding to promote attachment of primary neurons cells.

Rat primary cortical astrocytes (N7745100, Thermo Fisher Scientific) were maintained in Dulbecco’s modified Eagle’s medium high glucose (Sigma-Aldrich) supplemented with 10% fetal bovine serum and 1% penicillin/streptomycin (Fisher Scientific). Cells were seeded at a density of 20,000 cells per well in an xCELLigence E-plate 16 (Agilent, Santa Clara, CA, USA). No coating was used to promote the attachment of primary astrocytes.

When indicated, apoptosis was induced in SH-SY5Y cells, astrocytes, and co-culture of neurons and glial cells by adding As_2_O_3_ directly in the culture media at the indicated concentration. Autophagy was induced in HCT116 cells by serum deprivation following replacement of the DMEM nutrient rich culture medium by Hanks’ Balanced Salt Solution (HBSS, Cat. No. 14025092; Thermo Fisher Scientific) containing 1 g/L Glucose and buffered with NaHCO_3_ (2.2 g/L) [25].

### 4.3. Cellular Impedance Assay

Cellular impedance measurement of global cellular activity was monitored in a humidified incubator at 5% CO_2_ and 37 °C using the xCELLigence apparatus (Agilent). Using this setup, changes induced in the local ionic environment at the electrode/solution interface yield an increase in electrode impedance. Changes in cell morphology and/or adhesion that modulate the physical contact between cells and electrodes are reflected in impedance changes. The change in impedance is reported as a dimensionless parameter called Cell Index (*CI*) according to Equation (1):

(1) $$CI=\frac{\mathrm{impedance}\;\mathrm{at}\;\mathrm{time}\;\mathrm{point}\;n\;–\;\mathrm{impedance}\;\mathrm{without}\;\mathrm{cells}}{\mathrm{nominal}\;\mathrm{impedance}}$$

As no commercial system allowed cellular impedance measurement under RF exposure, we adapted the xCELLigence apparatus to our requirement. Using a parallel electrical circuitry bridging the RF signal generator to the gold connectors of the xCELLigence plates, we simultaneously performed impedance measurement while delivering the RF signals (CW, GSM, Wi-Fi, UMTS, and LTE) to the attached cells. The numerical and experimental dosimetry and the setup of the exposure system formed by the xCELLigence and RF devices (xCELL-RF) were presented in detail in Garcia-Fernandez et al. (2016) [15]. We used the internal design of our xCELL-RF setup to deliver 1800 MHz RF signals at various input powers resulting in S.A.R.s of 5, 7.6, 11.3, and 24 W/kg.

**Figure 6:**
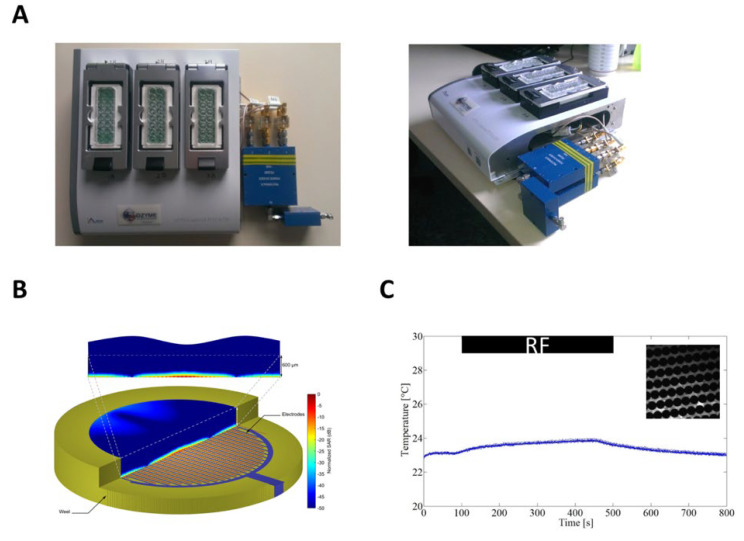
xCELL-RF system for simultaneous RF exposure and impedance measurements of cells. (**A**) xCELL-RF device with the RF connections to the dock. (**B**) Normalized S.A.R. distribution in the delivery system at 1800 MHz. For better readability, only half of the S.A.R. distribution and a vertical cut plane (top inset) are represented on the figure. (**C**) Experimental measurement of temperature elevation at the cell level using a S.A.R. of 240 W/kg, inset: example of picture acquired during temperature measurement.

A vector generator (SMBV100A, Rohde & Schwarz, Munich, Germany) delivering 1800 MHz RF signals was connected to a bidirectional coupler (ZGBDC30-372HP+, Mini-circuits, Brooklyn, NY, USA). The coupler was followed by a 2-way power splitter (PE2088, Pasternack, Irvine, CA, USA), and each way was connected to a 3-way power splitter (PE2090, Pasternack) which delivered the required powers to the xCELLigence Eplate (Figure 6A). The setup’s incident and reflected powers were monitored using two power sensors (N1921A, Agilent, Santa Clara, CA, USA) and a power meter (Agilent) connected to the bidirectional coupler. High values of RF electric field and S.A.R. are mainly localized in the proximity of the electrodes and within the cells layer, as attested by Finite Difference Time Domain (FDTD)-based numerical dosimetry (Figure 6B).

The temperature variation was measured using an optical probe (Luxtron, Santa Clara, CA, USA). The temperature at the cell level was increased by 0.5 °C when the culture dish was exposed at the high S.A.R. value of 240 W/kg (Figure 1C). This slight temperature increase associated with a high S.A.R. value is due to the rapid heating diffusion by the whole culture media that buffered the temperature increase. The temperature at the cell level during exposure to RF at 24 W/kg monitored in a separate experiment did not evidenced any detectable variation.

The E16 xCELLigence plates were prepared by adding cells in a complete medium (100 µL) as indicated in the cell culture section. When indicated, 100 µL of PBS buffer or a 2X ATO solution was added to the culture medium.

### 4.4. Impedancemetry Data Analysis

Four metrics were defined to characterize the possible effects of applied physical and chemical stimuli on the evolution of cell indexes.

Metric 1 (post-seeding plateau) is the median value of cell index in the 5 h preceding the moment of As_2_O_3_ injection. This metric highlights a possible effect of the exposures on the cell index before the injection of As_2_O_3_.

The three following metrics were calculated on normalized data, taking the time of the As_2_O_3_ injection as a reference point (with a relative value of 1). The metric 2 (peak) is the maximal CI variation following As_2_O_3_ injection. Metric 3 (T80) was calculated only for experiments with As_2_O_3_ injection and represents the time at which CI decreased by 80% following As_2_O_3_ injection. Finally, metric 4 (slope) is calculated only on experiments without As_2_O_3_ injection (chemical sham) and represents the slope of the cell index increase during the last 10 h of the experiments.

### 4.5. Exposure to RF and Holographic Image Acquisition

Digital holographic microscopy images were obtained using a HoloMonitor^®^ M4, (Phase Holographic Imaging AB (PHIAB), Lund, Sweden).

The RF exposure system was a tri-plate open transverse electromagnetic (TEM) cell allowing RF signals propagation [56]. Both TEM cell ground plates had apertures to allow for light propagation through the cell culture. Numerical modeling and simulations of aperture’s effect on cell exposure were performed as described previously [22,24]. A vector generator (SMBV100A, Rohde & Schwarz, Munich, Germany) connected to a 10-W amplifier (RF14002600-10, RFPA, Artigues-Près-Bordeaux, France) with around 40-dB gain was used to deliver 1800 MHz RF signals (CW and GSM) to the exposure system.

**Figure 7:**
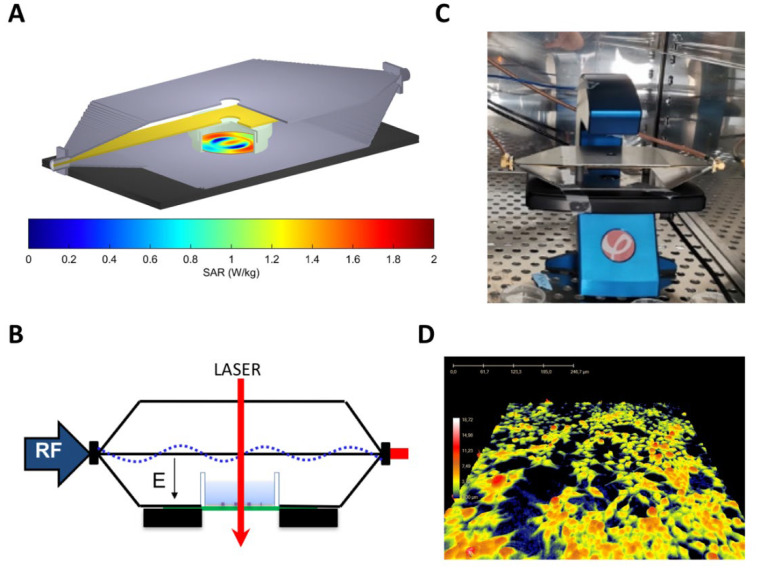
Experimental device for exposing cells to RF while acquiring digital holographic microscopic images. (**A**) Normalized S.A.R. distribution at the cell level in the delivery system at 1800 MHz. (**B**) Cross-section of the TEM cell in which a cell culture dish has been placed. The picture indicates the propagation of the RF electric field and the LASER path only at the center of the cell culture plate. (**C**) Photography of the TEM cell placed on the HoloMonitor M4 platform. A hole has been drifted in both ground planes and septum of the TEM cell to allow light to go through the cell culture. (**D**) Example of a digital holographic microscopy image of a SH-SY5Y cell culture. The color scale provides information on the height of the cells.

The effect of CW and GSM-modulated signals, using an 1800 MHz carrier wave, at S.A.R. of 1.5 and 6 W/kg was studied. Experimental dosimetry was carried out using temperature assessment measured with an optical probe (Luxtron). Numerical simulations with an in-house FDTD allowed the extraction of S.A.R. distribution at the cell layer level within the Petri dish exposed in the TEM cell (Figure 7A). The cell culture medium temperature increased by 0.5 ± 0.1 °C at 1.5 W/kg and 2 ± 0.1 °C at 6 W/kg during the first hour of exposure. The cell culture incubator temperature was decreased accordingly to maintain the cell culture medium temperature at 37 °C during RF exposure, as verified in a separate experiment using an optical probe (Luxtron). Cells were temperature equilibrated for several hours before the experiment started.

DHM images were acquired over a small area in the Petri dish center where the S.A.R. is homogenous (Figure 7B).

Twenty-four hours before exposure, cells were seeded at a density of 300,000 cells in a 35 mm petri dish and left in culture in a humidified incubator at 5% CO_2_ and 37 °C. On the day of the experiment, the Petri dishes’ lid was replaced by a HoloLid^TM^ (PHIAB). Next, cell culture dishes were placed in an exposure system within the HoloMonitor^®^ M4 platforms (PHIAB) (Figure 7C). Two identical setups were used for sham and RF-exposure conditions, but no RF was delivered to the sham condition’s cell culture.

A time-lapse movie was created using the software H-studio^TM^ (PHIAB) for both RF- and sham-exposure, with one capture taken every 5 min for up to 72 h in total in a humidified incubator at 5% CO_2_ and 37 °C (Figure 7D). The effect of CW and GSM-modulated signals (using an 1800 MHz carrier wave) at 1.5 and 6 W/kg was studied. Twenty-four hours after the start of the exposure phase, the culture medium was replaced with 4 mL HBSS medium without fetal bovine serum (serum deprivation) to trigger autophagy [57]. Average cell area, cell volume, and cell thickness were computed using the software H-studio^TM^.

### 4.6. DHM Data Analysis

Three metrics were used to analyze the DHM data. Metric 1 describes the median value of the signal in a 3 h interval before serum deprivation. Metric 2 is the median value of the signal during the 3 h after the media change. Metric 3 is the slope calculated by linear regression in a time interval corresponding to 40 to 60 h after the beginning of the RF exposure.

Metric 1 is calculated on the raw signal values. Metrics 2 and 3 are calculated on the normalized data, following normalization of the average cell area to 1 at time t = 24 h.

### 4.7. Statistics

Two samples comparisons were made using Wilcoxon signed-rank test. Multiple comparisons were made using Kruskal-Wallis tests and posthoc Conover tests. The significance results are all given at the alpha = 5% risk. The tools used for these analyses were the R language [58] and the following packages: tidyverse [59], FreqProf [60], stringi [61], and PMCMRplus [62].

Statistically significant differences between pairwise comparisons resulting from post hoc tests are labeled with letters (a, b, c...). This compact letter representation (CLD) is a method for correctly representing significance statements resulting from pairwise comparisons: for example, the graph with “ab” is not significantly different from a graph labeled with “a” or “b” but is statistically different from a graph labeled with “c” [63].

## Data Availability

Data are available upon request by writing to the corresponding author.

## Supplementary Materials

The following are available online at https://www.mdpi.com/article/10.3390/ijms23020658/s1.
