# Supplementary material for: Label-Free Study of the Global Cell Behavior during Exposure to Environmental Radiofrequency Fields in the Presence or Absence of Pro-Apoptotic or Pro-Autophagic Treatments
Source: Int J Mol Sci. 2022 Jan 8;23(2):658. doi: 10.3390/ijms23020658 (PMC8776001; doi:10.3390/ijms23020658)
Supplement: Supplementary file 1 [file ijms-23-00658-s001.zip › ijms-1476867-supplementary.pdf]

Supplementary Table S1: Number of replicate per experimental condition performed using the XCell-RF setup.

		Mock condition					As_2O_3				
	S.A.R. (W/kg) :	sham	5	7.6	11.3	24	sham	5	7.6	11.3	24
Astrocytes	CW	40	11	11	7	11	32	9	9	9	9
	GSM	32	8	8	8	8	32	8	8	8	8
	Wi-Fi	32	8	8	6	8	32	8	8	8	8
neurone / glial cells	CW	24	6	6	6	5	24	6	6	6	6
	GSM	24	6	6	6	6	24	6	6	6	6
SH-SY5Y	CW	4	4	4	4	4	7	4	4	4	4
	GSM	12	6	6	6	6	12	6	6	6	6
	Wi-Fi	20	9	8	7	7	12	8	8	8	8
	LTE	16	8	8	8	8	16	8	8	8	8
	UMTS	12	6	6	6	6	12	6	6	6	6
